# Conjunctival Pigmentation at the Sclerotomy Site following 23 G Vitrectomy with Silicone Oil Injection

**DOI:** 10.1155/2022/4978924

**Published:** 2022-11-29

**Authors:** Nazimul Hussain

**Affiliations:** ^1^Department of Ophthalmology, Mediclinic Parkview Hospital, Dubai, UAE; ^2^Department of Ophthalmology, Al Zahra Hospital, Sharjah, UAE

## Abstract

54-year-old gentleman undergoing 23 Gauge vitrectomy with silicone oil injection developing conjunctival pigmentation at one of the entry sites of trocar and cannula 1 week postoperative. The conjunctival tissue was biopsied at the time of silicone oil removal and correlated histopathological. Biopsy of the conjunctival tissue showed intracellular and stromal melanin pigments. This report highlights conjunctival pigmentation in suture less entry site and silicone oil as intraocular tamponade than earlier published reports of with or without intraocular gas tamponade.

## 1. Introduction

Conjunctival pigmentation can occur following traumatic scleral tear repair or following microincisional vitreous surgery [1–3]. Such pigmentations can also be congenital or acquired following melanoma, drug induced, or other systemic illnesses [4–7].

We present a case of conspicuous conjunctival pigmentation following 23 G suture vitrectomy with silicone oil injection in one of the entry sites.

## 2. Case Report

**Figure 1:**
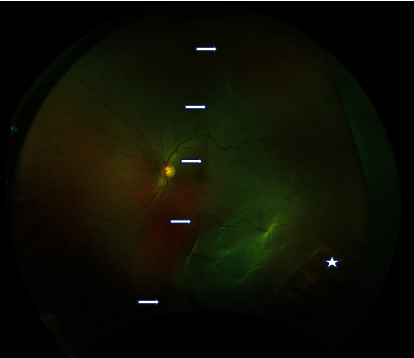
Wide-field fundus photo of left eye showing the temporal retinal detachment involving partially the macula (arrow) and lattice degeneration with retinal hole (Asterisk).

A 54-year-old gentleman presented to the retina clinic with complaints of sudden diminution of vision in the left eye noticed in the past 1 week. There is no significant past ocular or systemic history of illness. On examination, his distant visual acuity was uncorrected 6/6 in the right eye and counting fingers 2 meters in the left eye. Best corrected near vision was N6 in the right eye and < N36 in the left eye. Intraocular pressure was 14 mm Hg in the right eye and 11 mm Hg in the left eye. Anterior segment examination was unremarkable including the conjunctiva in both eyes except the lens showed early cortical changes in both eyes. Dilated fundus examination showed clear ocular media with attached retina and multiple lattice degeneration in the peripheral retina in the right eye. Left eye showed clear ocular media, temporal hemiretina detachment involving the macula, and peripheral lattice degeneration with retinal holes (Figure 1). Based on clinical examination, he was diagnosed as lattice degeneration in the right eye and rhegmatogenous retinal detachment in the left eye.

**Figure 2:**
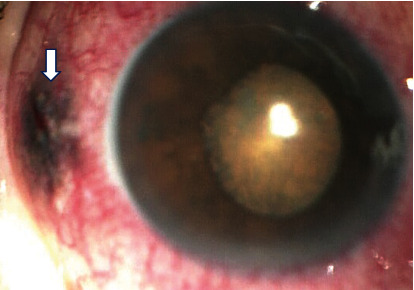
Showing conjunctival bluish black pigmentation of the conjunctiva (Arrow) in the superotemporal quadrant in the left eye.

After informed consent and discussion, he underwent barrage laser photocoagulation around the lattice degeneration in the right eye and 23 G vitrectomy, silicone oil injection, and endolaser in the left eye uneventfully. On the first postoperative day, anterior segment examination showed flat lids, mild congested conjunctiva, clear cornea, deep and quiet anterior chamber, mild dilated pupil, and cortical cataract changes in the left eye. Intraocular pressure was 15 mm Hg in the left eye. Fundus examination of the left eye showed clear media, attached retina, fresh laser marks in the periphery, and silicone oil in the vitreous cavity. An impression of stable status and reattached retina was made. On 1-week postoperative follow up, his retina status was stable, but anterior segment examination showed conjunctival pigmentation in the superotemporal quadrant at the site of trocar cannula entry. 3 months postoperative, his best corrected visual acuity was 6/18 in the left eye. Anterior segment examination in left eye showed conjunctival pigmentation in the superotemporal quadrant (Figure 2) and quiet conjunctiva, clear cornea, deep and quiet anterior chamber, and round and reacting pupil; lens showed early posterior subcapsular cataract with cortical cataract, and intraocular pressure was 16 mm Hg. Fundus examination showed mild hazy media, silicone oil in the vitreous cavity, attached retina, and peripheral laser scars in the left eye. An impression of secondary postsurgical conjunctival pigmentation, cataract, and reattached retina with silicone oil in the left eye was made. He then underwent conjunctival biopsy and silicone oil removal in the left eye.

**Figure 3:**
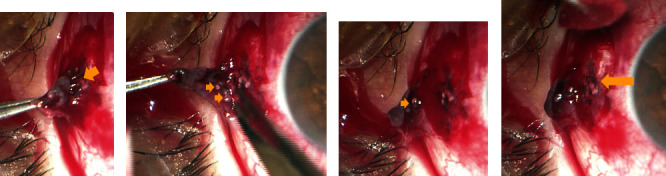
(a) Showing subconjunctival scarring (arrow), (b, c) subconjunctival silicone oil bubbles, and (d) dispersed pigmentation on the scleral surface .

**Figure 4:**
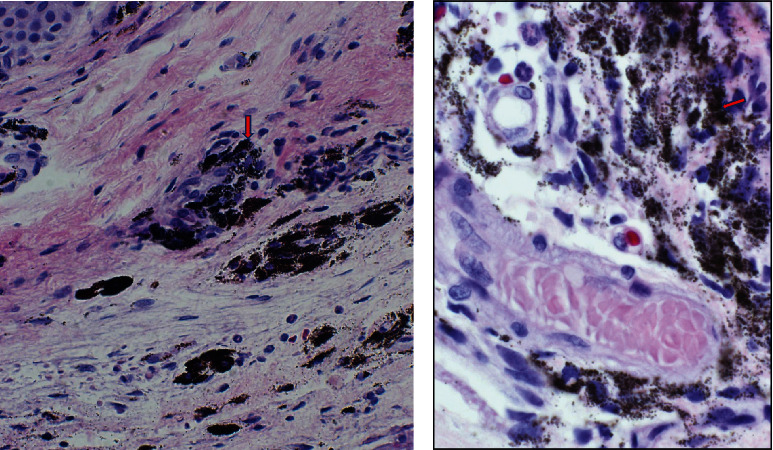
(a) Showing light microscopy and (b) higher magnification with intracellular pigments and stromal pigments (arrow).

**Figure 5:**
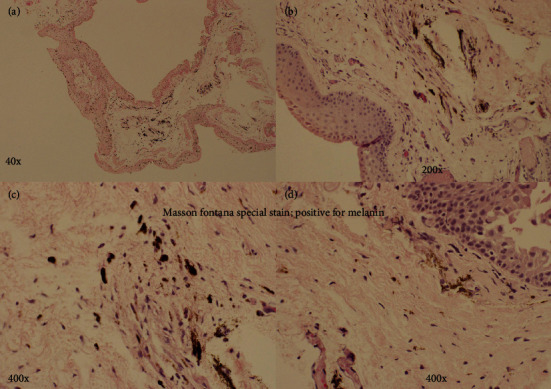
The Masson-Fontana special stain showing positive for melanin pigments.

Intraoperatively, the area of conjunctival pigmentation demonstrated extensive scarring with entrapped minute silicone oil bubbles within the subconjunctival scar. Dispersed light pigmentation was also seen on the scleral surface (Figures 3(a)–3(d)). The dissected conjunctival tissue was sent for Histopathological examination. Gross examination showed a wedge of conjunctival tissue measuring 0.3 cm. Microscopic examination showed intracellular and stromal infiltration of dark pigments seen in the stroma and intracellular (Figure 4). The Masson-Fontana special stain was positive for melanin pigments (Figure 5).

3 months post silicone oil removal, the patient underwent phacoemulsification with intraocular lens implantation in the left eye, and his visual acuity achieved 6/9, N6 in the left eye.

Informed consent and using patient data for research purposes was taken, and institutional review board permission was taken for research and publication of the case.

## 3. Discussion

Acquired conjunctival melanosis is seen secondary to many conditions including drug-induced, trauma or systemic disorders [3]. Smiddy et al. [3] have narrated a 34-year-old gentleman undergoing pars plana vitrectomy with fluid gas exchange for retinal detachment developing conjunctival pigmentation in the inferotemporal quadrant 6 weeks later. The pupil colour was brown, and he was Caucasian male. The surgery was performed with 20G instruments, and conjunctival pigmentation was attributed to cryotherapy applied before vitrectomy. They proposed that this could be due to reactivation of preexisting quiescent melanocytes, and the pigment distribution appears exogenous. They concluded that the source of melanin is probably from ruptured uveal and RPE cells treated by cryotherapy prior to vitrectomy.

Park et al. [2] have shown in their series of patients who underwent 23 G microincision vitreous surgery (MIVS) with fluid gas exchange and endolaser developing conjunctival pigmentation at the entry sites in 4.3% (8/185 patients) of patients. Interestingly, Park et al. have closed the entry sites at the end of surgery with a single releasable 8-0 polyamide suture. All patients showed conjunctival pigmentation within 7 days of surgery. Conjunctival pigmentation was observed in inferotemporal and superotemporal as well as superonasal entry sites. It was also observed that in eyes which had C3F8 tamponade, the surface area of pigmentation was larger than those eyes which did not have tamponade. The etiology of the cases was mostly proliferative diabetic retinopathy and one eye with rhegmatogenous retinal detachment. Multivariate logistic regression analysis confirmed that C3F8 tamponade (OR 9.4; 95% CI, 1.9-44.5; *P* = 0.005) was associated with conjunctival pigmentation after 23G MIVS in the study. However, performing complete vitrectomy as a responsible factor cannot be ruled out. It may increase the possibility of pigment exterior pigment fall out.

Our case showed conjunctival pigmentation in the superotemporal quadrant near to the entry site at 1 week. A 23G MIVS trocar cannula was used, and no sutures were applied at the end of the procedure. Histopathology and special stain for melanin have shown that the melanin pigments were both in the stroma and intracellular suggesting non-preexisting pigments. Besides, as shown in Figure 3, minute silicone oil bubbles enmeshed in the subconjunctival scar along with pigmentation suggest its origin from the uveal tissue at the entry site. However, silicone oil infiltration was not seen in the biopsied conjunctival tissue. Possible slow gradual leakage of in the initial time of healing of the wound has resulted in silicone oil bubbles and visibility of conjunctival pigmentation at 1 week.

Several studies [8–11] have shown that the migration of silicone oil bubbles in the subconjunctival tissue initiates chronic inflammatory process evident by presence of histiocytes and multinucleated lymphocytes. This suggests granulomatous inflammatory reaction to silicone oil. This chronic inflammatory process causes scar, and subconjunctival trapped silicone oil droplets. In our case, silicone oil droplets were seen in the subconjunctival tissue entrapped in the scar tissue.

Uveal pigment migration mostly happens in the initial period of minimal leakage along with silicone oil migration from entry site as evident in intracellular and intrastromal melanin pigments on histology. Chronic inflammation causes the subconjunctival scarring and heals the leaking entry site.

The highlight of the present case in contrast to the published reports is the use of silicone oil tamponade and not suturing the entry sites in 23G MIVS. The patient has brown eyes and middle eastern ethnicity.

In conclusion, the present case suggests that postoperative acquired conjunctival melanosis can occur in a patient undergoing 23G MIVS, and the possible use of sutures at the entry site can reduce the incidence lower than 4.3% as published earlier. Use of finer gauge MIVS like 27G can reduce such occurrence, but checking for leakage at the entry site seems mandatory especially in brown eyes.

## Data Availability

All data enumerated in the manuscript.
